# Mechanism of cystogenesis in nephrotic kidneys: a histopathological study

**DOI:** 10.1186/1471-2369-15-3

**Published:** 2014-01-08

**Authors:** Marijan Saraga, Katarina Vukojević, Vjekoslav Krželj, Zvonimir Puretić, Ivana Bočina, Merica Glavina Durdov, Stefanie Weber, Bernd Dworniczak, Danica Galešić Ljubanović, Mirna Saraga-Babić

**Affiliations:** 1 Department of Paediatrics, University Hospital in Split, Split, Croatia; 2 Department of Anatomy, Histology and Embryology, School of Medicine, University of Split, Split, Croatia; 3 Department of Dialysis, University Hospital Centre Zagreb, Zagreb, Croatia; 4 Department of Biology, University of Split, Split, Croatia; 5 Department of Pathology, University Hospital in Split, Split, Croatia; 6 Division of Pediatric Nephrology, University Children’s Hospital Essen, Essen, Germany; 7 Department of Human Genetics, University Münster, Münster, Germany; 8 Department of Pathology, University Hospital in Zagreb, Zagreb, Croatia

**Keywords:** Nephrotic kidney, Cystogenesis, CNF, FSGS, Cell proliferation, Primary cilia, Apoptosis

## Abstract

**Background:**

Nephrotic syndrome (NS) is pathological condition characterized by heavy proteinuria. Our study investigates hypothesis that change in cell proliferation of proximal tubules influences primary cilia structure and function and promotes cystogenesis in congenital nephrotic syndrome of the Finnish type (CNF) and focal segmental glomerulosclerosis (FSGS).

**Methods:**

CNF kidneys were analyzed genetically. Proliferation (Ki-67), apoptosis (caspase-3), and primary cilia (α-tubulin) length and structure were analyzed immunohistochemically and ultrastructurally in healthy, CNF and FSGS kidneys. Cyst diameters were measured and correlated with proliferation index.

**Results:**

Proximal tubules cells of healthy kidneys did not proliferate. In nephrotic kidneys, tubules with apparently normal diameter covered by cuboidal/columnar epithelium (PTNC) contained 81.54% of proliferating cells in CNF and 36.18% in FSGS, while cysts covered with columnar epithelium (CC) contained 37.52% of proliferating cells in CNF and 45.23% in FSGS. The largest cysts, covered with squamous epithelium (CS) had 11.54% of proliferating cells in CNF and 13.76% in FSGS. Increase in cysts diameter correlated with changes in proliferation index, tubular cells shape, primary cilia formation and appearance of apoptotic cells.

**Conclusions:**

We present a novel histopathological data on the structure and possible changes in function of tubular cell in NS kidneys during cystogenesis. We suggest existence of common principles of cystogenesis in CNF and FSGS kidneys, including serious disturbances of tubular cells proliferation and apoptosis, and faulty primary cilia signaling leading to deterioration of proteinuria in NS kidneys.

## Background

The nephrotic syndrome (NS) is pathological condition characterized by several components, including proteinuria. In healthy kidneys, proteins are mainly reabsorbed in proximal tubules by receptor-mediated endocytosis [1]. In NS, the most often cause of renal failure is focal segmental glomerulosclerosis (FSGS), which results from interplay of genetic and external factors. Histologically, FSGS is characterized by podocyte depletion, segmental glomerular scaring and glomerular epithelial proliferation [2]. Another type of NS is congenital nephrotic syndrome of the Finnish type (CNF), a glomerular disease caused by mutation of NPHS1 gene that encodes protein nephrin, localized on the slit diaphragm [3]. In CNF, patients show massive proteinuria already *in utero*[4]. Although histological lesions in CNF are described as un-specific, dilations of proximal tubules with microcyst formation are most often found [5]. Other hereditary conditions are also associated with renal cysts, including ciliopathies - a group of diseases caused by genetic mutations of proteins residing on the primary cilia [6], a specialized cell surface organelle which coordinates cell proliferation, differentiation and apoptosis [7-9]. Ciliary microtubular organization enables intraflagelar transport (IFT) and transfer of information both in and out of the cell. Faulty cilia signaling in some cases leads to cysts formation [10]. By now, little attention has been paid to primary cilia in CNF, as mutated nephrin has not been associated to cilia function.

During normal kidney development, morphogenesis and function of primary cilia correlated with proper tubular cells proliferation and apoptosis [11], and with periodical appearance of primary cilia on the surfaces of tubular cells between cell cycles [12]. While during post-natal life primary cilia remained on the surfaces of tubular cells [12], they disappeared in rat podocytes already during development [13]. By now, investigations on the cystogenesis in CNF kidneys have not been connected neither to abnormal cell proliferation and apoptosis [4], nor to the primary cilia abnormalities but to the cell dedifferentiation [4].On the other hand, enhanced cell turnover was found in multicystic dysplastic kidneys [14].

In the present study we analyzed the ultrastructural and immunohistochemical characteristics of CNF tubular cells during cystogenesis, and compared them to tubular cells in FSGS and in normal kidneys. We present a novel data on tubular cell pathology in NS kidneys and suggest that the disturbed proliferation and apoptosis, and associated changes of primary cilia structure and function might be involved in cystogenesis of CNF and FSGS kidneys, and possibly influence clinical presentation of NS.

## Methods

### CNF patient –clinical diagnosis

CNF patient from non-consanguineous parents was reanimated because of muscular hypotonia and decreased vitality. He became swollen due to massive proteinuria within the first week of life, and developed all clinical and laboratory signs of CNF, including characteristic ultrasonographic picture of kidneys [15]. Patient developed several sepses and one thrombotic episode during the first year of life. He received supplementary therapy, and was treated with antibiotics. Due to massive proteinuria, he underwent nephrectomy at the age of 12 and 22 months and CAPD was initiated.

### Genetic analysis

Genomic DNA was isolated from peripheral blood leukocytes by standard laboratory methods. Mutational analysis was performed for *NPHS1* [NCBI AccN° NG_013356.1] by direct sequencing of all coding exons and exon-intron boundaries, and a homozygous missense mutation was indentified (c.1096A > C; p.Ser366Arg) in *NPHS1* gene [16].

### FSGS patients- clinical and pathological data

All together four patients were diagnosed clinically and histopathologically (Table 1).

**Table 1:** Clinical and histopathological characteristics of FSGS kidneys

**Name**	**Age of the onset**	**Maximal level of proteinuria**	**Renal function**	**Number of glomeruli per biopsy or nephrectomy**	**Globally sclerotic glomeruli (%)**	**Segmentally sclerotic glomeruli (%)**	**Type of lesion**
B.D.	6 y	50 g/day	ESRD	100	80	10	NOS
S.I.	1 y 4 mo	8 g/day	Normal	17	29	0	NOS
D.Đ.	10 y	32 g/day	CKD grade III	6	16.6	66.6	NOS
T.S.D.	1 y	4 g/day	Normal	12	8.3	25	cellular

NOS = not other specified; ESRD = end stage renal disease; CKD = chronic kidney disease.

### Immunohistochemistry and immunofluorescence

The kidney tissues were collected with permission of the Ethical and Drug Committee of the University Hospital in Split, Croatia in agreement with the Helsinki Declaration (Class: 003-081/11-03/0005, No: 2181-198-03-04/10-11-0024).

Healthy kidney tissues were taken at autopsy from a 2-year-old child during diagnostic procedure for the cause of death. From CNF patient (2 kidneys) and from 4 patients with FSGS kidney tissue was taken during diagnostic procedure following nephrectomy or kidney biopsy. Tissue pieces were fixed with 4% paraformaldehyde in phosphate buffer saline (PBS), embedded in paraffin, serially sectioned, deparaffinized and processed as described previously [12]. Sections for diaminobenzidine (DAB) staining were incubated with the mouse monoclonal antibody [6-11B-1] to acetylated α-tubulin (1:800, ab24610, abcam, Cambridge, UK) and nuclei were counterstained with haematoxylin [12]. Images from 142 sections were captured with digital camera.

For immunofluorescent staining, the rabbit anti-Ki-67 antigen (1:100, AB9260; Chemicon, Temecula, CA), rabbit anti-human/mouse active caspase-3 antibody (1:800; AF835, R&D Systems, Minneapolis, Minn., USA) and mouse monoclonal (6-11B-1) acetylated alpha-tubulin were used. Secondary antibodies were used at 1:300 dilutions: Rhodamine (AP124R; Jackson Immuno Research Lab, West Grove, PA) and Alexa fluor 488 donkey anti-rabbit (508205, Invitrogen, Oregon, USA). Nuclei were counterstained with DAPI and cover-slipped. Controls for specificity of staining, included omitting of the primary antibody from the staining procedure. Sections were examined by using a fluorescence microscope (Olympus BX61, Tokyo, Japan) equipped with digital camera (DP71).

### Light and electron microscopy

Kidney tissue pieces were dissected and processed as described previously [12]. Semi-thin sections, 1 μm thick were stained with toluidine blue; adjacent ultrathin sections were stained with uranyl acetate and lead citrate [12]. Electron micrographs were obtained with a Jeol 1200 EX microscope.

### Quantification and statistical analysis

Tissues pieces were analyzed using immunohistochemical methods for detection of primary cilia (α-tubulin) and cell proliferation (Ki-67). The cilia length and diameters of proximal tubules and cysts in CNF and FSGS were measured by image analysis using Olympus Cell software. Proliferation index was defined as the percentage of total number of Ki-67- positive cells in the proximal tubules, CNF and FSGS cysts. Diameters of proximal tubules and cysts were correlated (Pearson correlation analysis) with proliferation index. Data were analysed by the Kruskal-Wallis and Dunn's post hoc test and expressed as mean ± SD. Significance was accepted at p < 0.05.

## Results

### Light microcopic and electronmicroscopic diagnosis of NS

#### Haematoxylin and eosin

CNF: proximal tubules were partly cystically dilated, covered with epithelium showing hyaline degeneration or atrophy. Tubular lumina contained desquamated epithelia, eosinophilic fluid or hyaline casts, fibrosis with moderate mononuclear infiltration characterized the interstitium. Ultrastructurally, podocytes displayed loss of cell processes.

FSGS: showed different degrees of segmental glomerular sclerosis, interstitium infiltrations, tubular atrophy and dilations, at places forming proximal tubules cysts. Ultrastructurally, podocytes showed depletion of foot processes.

#### Immunohistochemical staining to anti-α-tubulin and DAB (diaminobenzidine) and cilia length measuring

**Figure 1:**
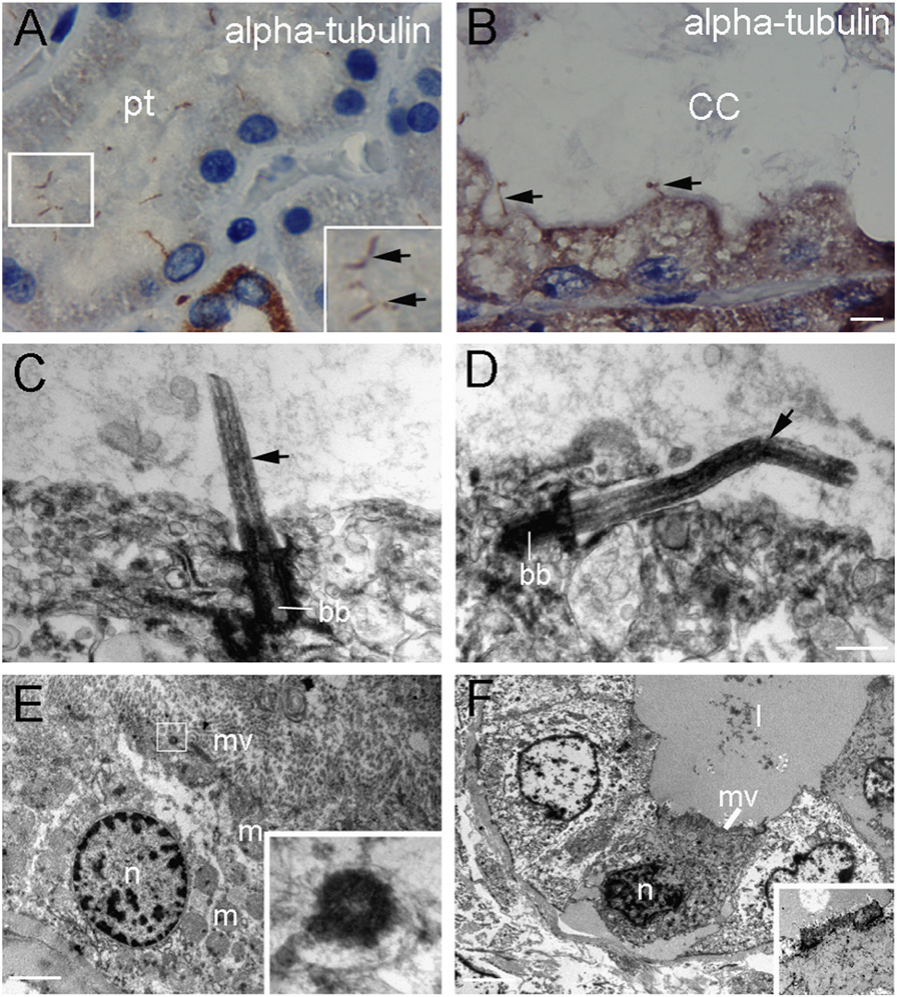
**Immunohistochemistry and electron microscopy of healthy and CNF kidneys: A**. Cortex of healthy kidneys with normal primary cilia (arrows in square) of proximal tubules (pt). **B**. CNF cysts of proximal tubules with columnar epithelium (CC) containing short and curly cilia (arrows). Immunostaining to α-tubulin, DAB and haematoxylin, scale bar 25 μm. **C**. Normal tubular primary cilia (arrow). **D**. “Broken” tubular primary cilia (arrow) in CNF cyst. Basal body (bb). Scale bar 0.1 μm. **E**. Ultrastructure of healthy tubular cell: oval nucleus (n), mitochondria (m), microvilli (mv) and basal body (bb) (square). Scale bar 1.2 μm. **F**. Ultrastructure of apoptotic cell in CNF cyst: dilated lumen (l) with colloid-like content, apoptotic cell with picnotic nucleus (n), reduced microvilli (mv)(square) and absence of cilia. Scale bar 1 μm.

In healthy kidneys, α-tubulin stained primary cilia of proximal and distal tubules and Bowman’s capsule of normal kidneys (Figure 1A).

In CNF (Figure 1B) and FSGS kidneys absence of primary cilia or distorted cilia were detected in the proximal tubules microcysts, while extremely long (8.41 ± 1.3 μm - 9.59 ± 1.6 μm long) cilia characterized distal/collecting tubules segment.

#### Electron microscopy

Primary cilia of healthy kidneys were perpendicular to the tubular cells surface (Figure 1C), while they appeared “broken” and aligned parallel to the cell surface in CNF (Figure 1D). Tubular cells of healthy kidneys had oval nuclei, abundant mitochondria and numerous microvili, and basal bodies or well developed cilia (Figure 1E). In CNF, tubular cells displayed reduction in apical microvili and cell height, while lumen contained colloid deposit. Shrinkage of nuclei and detachment of basal cell membrane characterized dying cells (Figure 1F).

#### Immunofluorescent staining to Ki-67, DAPI and α-tubulin, and statistical analysis of proliferation index and diameters of proximal tubules and cysts

**Figure 2:**
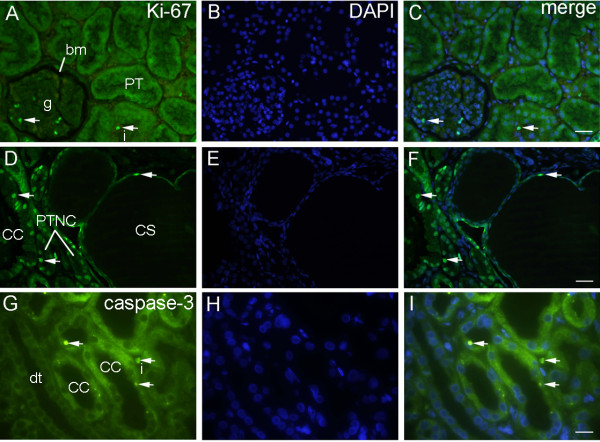
**Double immunofluorescent staining to Ki-67, caspase-3 and DAPI of healthy and CNF kidneys: A**. Healthy kidney cortex shows Ki-67-positive proliferating cells (arrows) in glomeruli (g), Bowman’s membrane (bm) and interstitium (i), but not in the proximal tubules (pt) **B**. Nuclear DAPI stain. **C**. Merging of A + B shows green proliferating and blue non-proliferating cell nuclei. Immunostaining to Ki-67 and DAPI, scale bar 25 μm **D**. CNF proximal tubules cysts of different diameters: apparently normal tubules (PTNC), cysts with simple squamous epithelium (CS) or cuboidal epithelium (CC) displaying numerous Ki-67- positive cells (arrows) **E**. Nuclear DAPI stain. **F**. Merging of D + E shows green Ki-67-positive cells (arrows) and blue non-proliferating cells in epithelium and interstitium. Immunostaining to Ki-67 and DAPI, scale bar 25 μm **G**. CNF kidneys show apoptotic caspase-3-positive cells (arrows) in dilated proximal (PTC), distal tubules (DT), and interstitium (i). **H**. Nuclear DAPI stain. **I**. Merging of G + H shows green apoptotic nuclei (arrows) and blue non-apoptotic nuclei. Immunostaining to caspase-3 and DAPI, scale bar 10 μm.

Healthy kidney: when stained with Ki-67 marker, in contrast to glomerules and interstitial cells, tubular cells showed no proliferation (Figure 2A-C).

**Figure 3:**
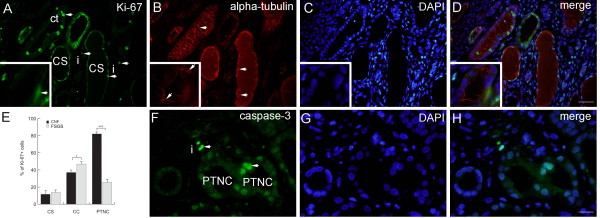
**Double immunofluorescent staining of FSGS kidneys to Ki-67, α-tubulin and DAPI or double immunofluorescent staining to caspase-3 and DAPI. A**. FSGS kidneys: cysts with simple squamous epithelium (CS) filled with colloid content, interstitium (i) and collecting tubules (ct) contain Ki-67-positive cells (arrows)(square). **B**. α-tubulin visualizes short cilia (arrows) in the cysts and extremely long cilia in collecting tubules (arrows)(square). **C**. Nuclear DAPI stain. **D**. Merging of A + B + C shows relationships between proliferating cells (green) and cilia (red). Immunostaining to Ki-67, α-tubulin and DAPI; scale bar 10 μm. **E**. Distribution of Ki-67 positive cells (%) in CNF and FSGS nephrotic syndrome. Cysts of proximal tubules with simple squamous epithelium (CS) or simple cuboidal epithelium (CC), proximal tubules with apparently normal simple cuboidal/columnar epithelium (PTNC). Data are shown as mean ± SD. Significant differences (Kruskal–Wallis) indicated by ***p < 0.0001. **F**. FSGS kidneys: caspase-3-positive apoptotic cells (arrows) in proximal tubules (PTNC) and interstitium (i). **G**. Nuclear DAPI stain. **H**. Merging of F + G shows green caspase-3- positive apoptotic nuclei and blue non-apoptotic nuclei. Immunostaining to caspase-3 and DAPI, scale bar 10 μm.

CNF: tubules with apparently normal diameter and simple cuboidal/columnar epithelium (PTNC) (diameter 12 ± 1.4 μm) contained 81.54% of Ki-67-positive cells, while tubular cysts with columnar epithelium (CC) (diameter 21 ± 1.8 μm to 54 ± 1.1 μm) contained 37.52% (Pearson r  =  0.9; *p*  =  0.0001), suggesting positive correlation between proliferation index and tubular diameter. The largest cysts with squamous epithelium (CS) (diameter 73 ± 2.1 μm) contained only 11.54% (Pearson r  =  -0.9; *p*  =  0.0001) of Ki-67-positive-cells (Figure 3E), suggesting a negative correlation between proliferation and cysts diameter. The increase in tubular diameter was associated with changes in cell shape, from columnar to squamous (Figure 2D-F).

FSGS: tubules with PTNC contained 36.18% of Ki-67-positive cells. In CC cysts their number increased to 45.23%, while in CS cysts decreased to 13.76% (Figure 3E). In CS cysts the primary cilia were short, while in distal/collecting tubules they were extremely long (Figure 3A-D).

***Immunofluorescent staining to caspase-3 and DAPI*** disclosed presence of caspase-3- positive cells in dilated tubules and cysts of proximal and distal tubules, and in the interstitium of both CNF (Figure 2G-I) and FSGS kidneys (Figure 3F-H).

## Discussion

Our ultrastructural and immunohistochemical analysis of CNF and FSGS kidneys disclosed that cystogenesis in proximal tubules was associated with increased cell proliferation, apoptosis and changes of primary cilia on the surfaces of tubular cells. While in the largest cysts of CNF kidneys the primary cilia were completely missing or were short and distorted, in moderately dilated or apparently normal tubules they were 3–8 fold longer than in healthy kidneys. Increased proliferation found in CNF cysts coincided with decreased number of primary cilia, while the increased diameter of proximal tubules or tubular cysts was inversely proportional to proliferation and was accompanied by the reduced height of tubular cells. The same type of changes in tubular cells characterized FSGS nephrotic kidneys during cystogenesis, as well. However, we believe that differences that we found in the course of proliferation between CNF and FSGS kidneys might be attributed to the prenatal appearance of pathological changes in CNF versus their postnatal appearance in FSGS. Both in CNF and FSGS kidneys, deregulations of cell turnover were accompanied by apoptosis of tubular and mesenchymal cells, as also described in human kidney malformations associated with urinary tract obstruction [14].

Experimental studies on kidney primary cilia confirmed association of primary cilia dysfunction and cystogenesis [8]. Thus, deleting of cilia assembly gene IFT20 prevented cilia formation and promoted rapid postnatal cystogenesis [17], while disturbed IFT resulted in a variety of disorders, including polycystic kidney disease [18]. In some cases, disturbed Wnt signaling, which mediates planar cell polarity (PCP), caused renal cystogenesis [19]. Abnormally short or extensively long cilia were found in human juvenile cystic kidney disease [20] and meckel syndrome, as well [21]. Investigations of ciliogenesis during normal human kidney development, described association of primary cilia lengthening with differentiation of tubular cells, apico-basal cell polarity and proper lumen formation. [12]. Similar to described findings in CNF and FSGS kidneys, increased cilium lengthening following ischemia-reperfusion injury characterized distal/collecting tubules segments [22]. Such downstream changes of primary cilia along the affected nephron might represent a compensatory process associated with loss of cilia in the cysts of proximal tubules. We suggest that described alteration of primary cilia number, structure or orientation might diminish the overall quality and quantity of tubular cells signaling, leading to compensatory growth of cilia in distal/collecting tubules segments in effort to increase signaling and preserve function of the damaged nephron. In Ofd1 syndrome, initially normally formed primary cilia disappeared during cystogenesis, suggesting secondary nature of cilia changes [23]. Recent studies on kidney cystogenesis pointed to significant influence of the extracellular milieu on modulation of cilia signaling, which led to deregulation of cell proliferation and cell differentiation [10]. We speculate that the described apoptosis and malfunction of proximal tubular cells during cytogenesis might cause significant deterioration of protein re-absorption in the affected kidneys. Consequently, the urine of CNF and FSGS kidneys becomes overloaded with proteins and therefore milieu for primary cilia signaling becomes further deteriorated.

## Conclusions

In conclusions, our study on CNF and FSGS tubular cells during cystogenesis revealed serious disturbances of cells proliferation, apoptosis and primary cilia formation, implying existence of general principle of kidney cystogenesis, independently of its cause. We suggest that cystogenesis in nephrotic kidneys starts with increased proliferation and apoptosis leading to disturbed lumen formation and damage of primary cilia. *Vice versa*, alterations of primary cilia structure or function caused by increased protein content within the cysts might lead to disturbed proliferation, differentiation and apoptosis of tubular cells in proteinuric kidneys.

## Abbreviations

NS: Nephrotic syndrome; CNF: Congenital nephrotic syndrome of the finnish type; FSGS: Focal segmental glomerulosclerosis; PBS: Phosphate buffer saline, DAB: diaminobenzidine; PCP: Planar cell polarity; IFT: Intraflagelar transport.

## Competing interest

There is no competing of interest regarding our manuscript.

This work was supported by the Ministry of Science, Education and Sports of the Republic of Croatia (grant no. 021-2160528-0507).

## Authors’ contributions

MS: Conception and design of study, acquisition and analysis of data, drafting and revision of manuscript. KV: Acquisition and analyzing of data, drafting and revision of the manuscript, statistical analysis. VK: Data collection, revision of manuscript. ZP: Data collection, revision of manuscript. IB: Data analysis, performing of electron microscopy, revision of manuscript. MGD: Data analysis, revision of manuscript. SW: Data analysis, performing of genetic analysis, revision of manuscript. BD: Data analysis, performing of genetic analysis, revision of manuscript. DGLJ: Data analysis, performing of electron microscopy, revision of manuscript. MSB: Conception and design of study, data analysis, drafting and revision of manuscript. All authors read and approved the final manuscript.

## Pre-publication history

The pre-publication history for this paper can be accessed here:

http://www.biomedcentral.com/1471-2369/15/3/prepub

## References

[B1] Keddis M Karnath B The nephrotic syndrome Revie of Clinical Signs 2007 38 25 30

[B2] D'Agati VD Pathobiology of focal segmental glomerulosclerosis: new developments Curr Opin Nephrol Hypertens 2012 21 3 243 250 10.1097/MNH.0b013e32835200df 22357339

[B3] Done SC Takemoto M He L Sun Y Hultenby K Betsholtz C Tryggvason K Nephrin is involved in podocyte maturation but not survival during glomerular development Kidney Int 2008 73 6 697 704 10.1038/sj.ki.5002707 18046313

[B4] Haltia A Solin ML Holmberg C Reivinen J Miettinen A Holthofer H Morphologic changes suggesting abnormal renal differentiation in congenital nephrotic syndrome Pediatr Res 1998 43 3 410 414 10.1203/00006450-199803000-00017 9505282

[B5] Niaudet P Genetic forms of nephrotic syndrome Pediatr Nephrol 2004 19 12 1313 1318 10.1007/s00467-004-1676-9 15503167

[B6] Waters AM Beales PL Ciliopathies: an expanding disease spectrum Pediatr Nephrol 2011 26 7 1039 1056 10.1007/s00467-010-1731-7 21210154 PMC3098370

[B7] Eggenschwiler JT Anderson KV Cilia and developmental signaling Annu Rev Cell Dev Biol 2007 23 345 373 10.1146/annurev.cellbio.23.090506.123249 17506691 PMC2094042

[B8] D'Angelo A Franco B The primary cilium in different tissues-lessons from patients and animal models Pediatr Nephrol 2010 26 5 655 662 20890766 10.1007/s00467-010-1650-7

[B9] Satir P Pedersen LB Christensen ST The primary cilium at a glance J Cell Sci 2010 123 499 503 10.1242/jcs.050377 20144997 PMC2818190

[B10] Gascue C Katsanis N Badano JL Cystic diseases of the kidney: ciliary dysfunction and cystogenic mechanisms Pediatr Nephrol 2011 26 8 1181 1195 10.1007/s00467-010-1697-5 21113628 PMC3640323

[B11] Carev D Krnic D Saraga M Sapunar D Saraga-Babic M Role of mitotic, pro-apoptotic and anti-apoptotic factors in human kidney development Pediatr Nephrol 2006 21 5 627 636 10.1007/s00467-006-0057-y 16568307

[B12] Saraga-Babic M Vukojevic K Bocina I Drnasin K Saraga M Ciliogenesis in normal human kidney development and post-natal life Pediatr Nephrol 2012 27 55 63 10.1007/s00467-011-1941-7 21688189

[B13] Ichimura K Kurihara H Sakai T Primary cilia disappear in rat podocytes during glomerular development Cell Tissue Res 2010 341 1 197 209 10.1007/s00441-010-0983-7 20495826 PMC2898502

[B14] Woolf AS Welham SJ Cell turnover in normal and abnormal kidney *development* Nephrol Dial Transplant 2002 17 Suppl 9 2 4 12386272 10.1093/ndt/17.suppl_9.2

[B15] Saraga M Jaaskelainen J Koskimies O Diagnostic sonographic changes in the kidneys of 20 infants with congenital nephrotic syndrome of the Finnish type Eur Radiol 1995 5 49 54 10.1007/BF02343261

[B16] Lenkkeri U Mannikko M McCready P Lamerdin J Gribouval O Niaudet PM Antignac CK Kashtan CE Homberg C Olsen A Structure of the gene for congenital nephrotic syndrome of the finnish type (NPHS1) and characterization of mutations Am J Hum Genet 1999 64 1 51 61 10.1086/302182 9915943 PMC1377702

[B17] Johanssen S Fassnacht M Brix D Koschker AC Hahner S Riedmiller H Allolio B Adrenocortical carcinoma. Diagnostic work-up and treatment Urologe A 2008 47 2 172 181 10.1007/s00120-007-1578-0 18030443

[B18] Yoder BK Hou X Guay-Woodford LM The polycystic kidney disease proteins, polycystin-1, polycystin-2, polaris, and cystin, are co-localized in renal cilia J Am Soc Nephrol 2002 13 10 2508 2516 10.1097/01.ASN.0000029587.47950.25 12239239

[B19] Simons M Gloy J Ganner A Bullerkotte A Bashkurov M Kronig C Schermer B Benzing T Cabello OA Jenny A Inversin, the gene product mutated in nephronophthisis type II, functions as a molecular switch between Wnt signaling pathways Nat Genet 2005 37 5 537 543 10.1038/ng1552 15852005 PMC3733333

[B20] Sohara E Luo Y Zhang J Manning DK Beier DR Zhou J Nek8 regulates the expression and localization of polycystin-1 and polycystin-2 J Am Soc Nephrol 2008 19 3 469 476 10.1681/ASN.2006090985 18235101 PMC2391053

[B21] Tammachote R Hommerding CJ Sinders RM Miller CA Czarnecki PG Leightner AC Salisbury JL Ward CJ Torres VE Gattone VH 2nd Ciliary and centrosomal defects associated with mutation and depletion of the Meckel syndrome genes MKS1 and MKS3 Hum Mol Genet 2009 18 17 3311 3323 10.1093/hmg/ddp272 19515853 PMC2733821

[B22] Verghese E Weidenfeld R Bertram JF Ricardo SD Deane JA Renal cilia display length alterations following tubular injury and are present early in epithelial repair Nephrol Dial Transplant 2008 23 3 834 841 17962379 10.1093/ndt/gfm743

[B23] Zullo A Iaconis D Barra A Cantone A Messaddeq N Capasso G Dolle P Igarashi P Franco B Kidney-specific inactivation of Ofd1 leads to renal cystic disease associated with upregulation of the mTOR pathway Hum Mol Genet 2010 19 14 2792 2803 10.1093/hmg/ddq180 20444807 PMC2893811

